# Exploring the realm of soft matter biophysics: an early career perspective

**DOI:** 10.1042/ETLS20220110

**Published:** 2022-12-21

**Authors:** Natasha H. Rhys

**Affiliations:** Department of Physics, King's College London, London WC2R 2LS, U.K.

**Keywords:** biophysics, early career, soft matter

## Abstract

This special issue of *Emerging Topics in Life Sciences* presents a selection of reviews that give insight into the vast array of research taking place in the fields of soft matter and biophysics, and where these two intersect. The reviews here cover the full range from the fundamentals of how biological systems may have assembled to how we can use this insight to develop and exploit new biomaterials for the future, all informed through the lens of the physical sciences. This issue has been both written and edited by early career researchers, highlighting the cutting-edge contributions that this generation of researchers is bringing to the field.

Interdisciplinarity in science is becoming increasingly more prevalent and in the understanding of biological systems much is to be gained from approaches and insights that have typically been classed as ‘the physical sciences’. This issue explores the areas of soft matter and biophysics, which can both use a physical sciences lens to understand biological systems more in depth.

Soft matter rose to prominence in the early 1990s when de Gennes was awarded the 1991 Nobel Prize in Physics *for discovering that methods developed for studying order phenomena in simple systems can be generalized to more complex forms of matter, in particular to liquid crystals and polymers* [1]. Soft matter itself encompasses materials that can be readily deformed [2], including but not limited to polymers, liquid crystals, colloids, surfactants, and biological systems. As a science, it sits at the interface between chemistry, physics, biology, and mathematics and explores the structure, properties, kinetics and self-assembly of these systems. Biophysics alternatively utilises principles and approaches typically used in physics to understand biological systems [3], including structure determination, microscopy, imaging, spectroscopy, scattering, and computational approaches. These techniques give rise to information at various length scales to help answer key biological questions. Both fields have progressed rapidly over the years and often overlap, addressing challenges not only in understanding the fundamental science behind biological systems but developing practical solutions for biomedical, pharmaceutical, food, and technological applications. The reviews presented in this issue span from the foundations of how biomolecules may have first formed and assembled, to how we can harness this knowledge to produce new materials for the future.

The role of soft matter research for understanding the origins of life is brought to the forefront in the review by Jia et al. [4]. They highlight the possible integral role nucleic acid liquid crystals may have in the formation of longer sequences relevant to the emergence of life, where they delve into both its plausibility and the environmental conditions for this to potentially occur. This is the bridge to how the ‘liquid crystal world’ [5] may be the precursor to the ‘RNA world’ hypothesis [6,7] which could allow functional polymers to develop.

A topic of long-term fascination is how biological molecules and systems can adapt to variety of conditions, including those considered extreme. Bhattacharya [8] explores this in their review on archaeal lipids, describing the ‘Lipid Divide’ [9] between these structures compared with lipids from bacteria and eukaryotes. This review covers how the structural differences these lipids possess impact their self-assembly and biophysical properties, which could allow archaea to thrive in a vast range of environments, including the human gut.

Aufderhorst-Roberts and Staykova [10] explore the progress being made in studying actin assemblies, with the actin cytoskeleton and its interaction with the plasma membrane in eukaryotes being pivotal to a variety of biomechanical functions. They delve into the role this plays for shape changes in cells, their mechanical reinforcement, and contraction processes, as well as the next steps to begin studying more complex processes.

The review by Meng [11] highlights how our increased knowledge of biological macromolecules has given rise to a deeper understanding of the advantageous properties they possess. This has led to the development of bioinspired and biomimetic materials across many applications, including novel biopolymer systems. From peptoids to polyelectrolyte complexes, this review explores how sequence and charge can be the key to the foundational understanding of synthetic polymer self-assembly and the formation of new materials.

Huang's paper [12] details the progress made in building increasingly complex vesicle structures. In particular, they examine the latest technical advances in the production of asymmetric unilamellar vesicles constructed from lipids and polymer–lipid mixtures. These can serve as more reliable models for natural cell membranes and Huang outlines both microfluidic and non-microfluidic techniques used to build these structures.

Finally, the review by Ghosh [13] describes the possible routes to designing artificial cells for various biotechnological applications. The review outlines the possible tools required to engineer these entities as well as the current applications they are applied to, including biosensing, drug delivery, and carbon fixation.

The articles in this issue have been solely written by, or have as corresponding author, an early career researcher. This special issue, therefore, sets to showcase the excellent and cutting-edge research being completed by the upcoming generation of scientists in this area and is the second issue of its kind to be published by a Portland Press journal [14]. It is hoped that this issue provides a taster of the vast array of research being completed in soft matter and biophysics by those who are set to shape this field in the future.

## References

[1] MLA style: The Nobel Prize in Physics. NobelPrize.org. Nobel Prize Outreach. https://www.nobelprize.org/prizes/physics/1991/summary/

[2] van der Gucht, J. (2018) Grand challenges in soft matter physics. Front. Phys. 22, 87 10.3389/fphy.2018.00087

[3] Mierke, C.T. (2020) The definition of biophysics: what exactly is biophysics? In Cellular Mechanics and Biophysics, Chap. 1, pp. 3–34 https://link.springer.com/chapter/10.1007/978-3-030-58532-7_1

[4] Jia, T.Z., Bellini, T., Clark, N. and Fraccia, T.P. (2022) A liquid crystal world for the origins of life. Emerg. Top. Life Sci. 6, ETLS20220081 10.1042/ETLS20220081 36373852

[5] Fraccia, T.P., Zanchetta, G., Rimoldi, V., Clark, N.A. and Bellini, T. (2015) Evidence of liquid crystal-assisted abiotic ligation of nucleic acids. Orig. Life Evol. Biosph. 45, 51–68 10.1007/s11084-015-9438-1 25975435

[6] Szostak, J.W. (2017) The narrow road to the deep past: in search of the chemistry of the origin of life. Angew. Chem. Int. Ed. 56, 11037–11043 10.1002/anie.201704048 28514493

[7] Robertson, M.P. and Joyce, G.F. (2012) The origins of the RNA world. Cold Spring Harb. Perspect. Biol. 4, a003608 10.1101/cshperspect.a003608 20739415 PMC3331698

[8] Bhattacharya, A. (2022) Self-assembly and biophysical properties of archaeal lipids. Emerg. Top. Life Sci. 6, ETLS20220062 10.1042/ETLS20220062 36377774

[9] Sojo, V. (2019) Why the lipid divide? Membrane proteins as drivers of the split between the lipids of the three domains of life. BioEssays 41, 1800251 10.1002/bies.201800251 30970170

[10] Aufderhorst-Roberts, A. and Staykova, M. (2022) Scratching beyond the surface - minimal actin assemblies as tools to elucidate mechanical reinforcement and shape. Emerg. Top. Life Sci. 6, ETLS20220052 10.1042/ETLS20220052 PMC9788373 36541184

[11] Meng, X. (2022) A mini-review on bio-inspired polymer self-assembly: single-component and interactive polymer systems. Emerg. Top. Life Sci. 6, ETLS20220057 10.1042/ETLS20220057 PMC9788401 36254846

[12] Huang, T. (2022) Assembly methods for asymmetric lipid and polymer-lipid vesicles. Emerg. Top. Life Sci. 6, ETLS20220055 10.1042/ETLS20220055 36533596

[13] Ghosh, B. (2022) Artificial cell design: reconstructing biology for life science applications. Emerg. Top. Life Sci. 6, ETLS20220050 10.1042/ETLS20220050 PMC9788358 36398710

[14] Gruszka, D.T. (2021) Biochemistry: one molecule at a time. Essays Biochem. 65, 1–3 10.1042/EBC20210015 33860798 PMC8056033

